# Neurometabolic changes in a rat pup model of type C hepatic encephalopathy depend on age at liver disease onset

**DOI:** 10.1007/s11011-023-01210-w

**Published:** 2023-05-06

**Authors:** Dunja Simicic, Veronika Rackayova, Olivier Braissant, Christian Toso, Graziano Oldani, Dario Sessa, Valérie A. McLin, Cristina Cudalbu

**Affiliations:** 1 CIBM Center for Biomedical Imaging, Lausanne, Switzerland; 2 Animal Imaging and Technology, Ecole Polytechnique Federale de Lausanne (EPFL), Lausanne, Switzerland; 3 Service of Clinical Chemistry, Lausanne University Hospital and University of Lausanne, Lausanne, Switzerland; 4 Division of Abdominal and Transplantation Surgery, Department of Surgery, Faculty of Medicine, Geneva University Hospitals, Geneva, Switzerland; 5 Faculty of Medicine, Hepato-pancreato-biliary Centre, Geneva University Hospitals, Geneva, Switzerland; 6 Swiss Pediatric Liver Center, Department of Pediatrics, Gynecology and Obstetrics, University of Geneva, Geneva, Switzerland

**Keywords:** Chronic liver disease, Type C hepatic encephalopathy, Neurodevelopment, Bile duct ligation, ^1^H MRS

## Abstract

Chronic liver disease (CLD) is a serious condition where various toxins present in the blood affect the brain leading to type C hepatic encephalopathy (HE). Both adults and children are impacted, while children may display unique vulnerabilities depending on the affected window of brain development.

We aimed to use the advantages of high field proton Magnetic Resonance Spectroscopy (^1^H MRS) to study longitudinally the neurometabolic and behavioural effects of Bile Duct Ligation (animal model of CLD-induced type C HE) on rats at post-natal day 15 (p15) to get closer to neonatal onset liver disease. Furthermore, we compared two sets of animals (p15 and p21-previously published) to evaluate whether the brain responds differently to CLD according to age onset.

We showed for the first time that when CLD was acquired at p15, the rats presented the typical signs of CLD, i.e. rise in plasma bilirubin and ammonium, and developed the characteristic brain metabolic changes associated with type C HE (e.g. glutamine increase and osmolytes decrease). When compared to rats that acquired CLD at p21, p15 rats did not show any significant difference in plasma biochemistry, but displayed a delayed increase in brain glutamine and decrease in total-choline. The changes in neurotransmitters were milder than in p21 rats. Moreover, p15 rats showed an earlier increase in brain lactate and a different antioxidant response. These findings offer tentative pointers as to which neurodevelopmental processes may be impacted and raise the question of whether similar changes might exist in humans but are missed owing to ^1^H MRS methodological limitations in field strength of clinical magnet.

## Introduction

Chronic liver disease (CLD) is a serious condition that can develop in adults or children. In CLD, various toxins present in high concentrations in the blood stream (e.g. bilirubin, ammonium, bile acids, inflammatory factors) affect the brain and lead to a neuropsychiatric disorder called type C hepatic encephalopathy (HE) (Jalan and Kerbert 2020). Both adults and children are impacted, and children may display unique vulnerabilities depending on the affected window of brain development (Caudle et al. 2010; McLin and D’Antiga 2022).

CLD is associated with neurocognitive deficits in adults, which are partially reversible, while the brain of children might be more sensitive to the consequences of CLD because of ongoing brain development and growth (Braissant et al. 2013; Felipo 2013; Semple et al. 2013). Indeed, there is growing evidence that neurocognitive impairment in children with CLD present before liver transplantation persists also after (Gilmour et al. 2010; Robertson et al. 2013; Ng et al. 2014; Sorensen et al. 2014; McLin and D’Antiga 2022). We know that children with urea cycle disorders exposed to hyperammonemia in infancy experience life-long neurocognitive consequences, in excess of what is seen in adults, suggesting that insults in childhood are potentially associated with severe outcomes (Enns 2008; McLin and D’Antiga 2022). Plasma ammonium concentrations in children with CLD are not as high as in urea cycle disorders. Nonetheless, the cumulative exposure to low grade hyperammonemia spanning several developmental windows on the brain of children with liver disease is increasingly accepted to be deleterious. However, very few studies on the developmental metabolic events in healthy children and patients with CLD have been performed to date (Foerster et al. 2009; Razek et al. 2014; Hanquinet et al. 2017; Srivastava et al. 2017). What more, the value of these studies in our understanding of the neurometabolic consequences of CLD in the developing brain is limited by the low magnetic fields at which they were performed. The lower spectral resolution of lower magnetic fields is an impediment to detecting all relevant metabolites, especially the separation between glutamine and glutamate.

We previously showed in an animal model of CLD, that rats who underwent bile duct ligation (BDL) at post-natal day 21 (p21) (still during a period of brain development) suffered from more pronounced changes in many brain metabolites (a stronger increase in brain glutamine, decrease of osmolytes, energy metabolites, neurotransmitters and antioxidants) compared to adult BDL rats (Braissant et al. 2019; Rackayova et al. 2020).

Many children develop liver disease at a younger age, before the age of 9 months (equivalent to the developmental stage of a rat brain at p21 (Workman et al. 2013)), leading to the very limitation of the p21 study (Rackayova et al. 2020). Therefore, we aimed to use our unique set of tools to study longitudinally the neurometabolic and behavioural effects of BDL on animals having developed disease at post-natal day 15 (p15) corresponding to ≈ 4 months old human brain (Workman et al. 2013) to get closer to neonatal onset liver disease. We used the experimental advantages of high field proton Magnetic Resonance Spectroscopy (^1^H MRS) to analyze the longitudinal changes of brain metabolites in vivo measured in the hippocampus of rats having undergone BDL at p15 together with plasma biochemical parameters and behavioural tests. The final goal was to compare the two sets of animals (p15 and p21 (Rackayova et al. 2020)) to begin to unravel whether the brain responds differently to CLD according to age onset, and therefore developmental window.

## Methods

### Study design

All animal experiments were conducted according to federal and local ethical guidelines, and the protocols were approved by the local Committee on Animal Experimentation for the Canton de Vaud, Switzerland (VD2761).

Eight (8) male Wistar pups underwent bile duct ligation (BDL) surgery and 8 male Wistar pups were sham operated at p15. One BDL pup was removed from the study as it recovered from surgery (plasma bilirubin decreased at normal values two weeks after surgery) leading to a total of 7 BDL pups entering the study. The progression of CLD and HE was monitored longitudinally in each animal until post-operative week 6. The BDL rat model is an animal model of type C HE recognized by the International Society for Hepatic Encephalopathy and Nitrogen Metabolism. In this model, biliary cirrhosis is associated with hyperammonemia, jaundice and portal hypertension (Butterworth et al. 2009; DeMorrow et al. 2021). Wistar dams and their male pups were obtained on postnatal day 10 or 11 (p10/p11) from Charles River laboratories (L’Arbresle, France). The days that preceded BDL surgery, an olfactory conditioning of the dams was performed by putting wadding soaked with disinfectant solution (used during the surgery), in order to get mothers used to the smell of the pups after the surgery. On day p15, BDL surgery was performed under isoflurane anaesthesia: the common bile duct (CBD) was isolated and ligated as previously described (Rackayova et al. 2016, 2020; Braissant et al. 2019). Sham animals underwent transverse laparotomy and mobilization of the CBD, also on day p15. After the surgery, the pups were returned to the cage with their mother only after they regained their motor skills and the ability to vocalize, the latter being important for interaction with the mother. The interaction between mothers and pups after the surgery was carefully monitored.

With the aim of putting our study in the context of human development, we used a previously published model for the extrapolation of brain developmental characteristics between mammalian species (Workman et al. 2013) http://translatingtime.org/translate). Nevertheless, mammalian species develop at different rates, particularly when it comes to neurodevelopmental processes and brain regions, so determining when all neurodevelopmental processes in rats translate in a specific time window in humans is complex and challenging (Erecinska et al. 2004). Here we estimated that the sensorimotor development and brain growth (myelination, neurogenesis, axonal growth), specifically of the limbic system of the rat at p15 (day of BDL) corresponds to ≈ 4 months old human. The results from this study were compared to the group of rats that underwent BDL surgery at p21 (N = 12) (Rackayova et al. 2020), which corresponds to ≈ 9 months old human (Workman et al. 2013). Note that p21 and p15 operated BDL rats were scanned at the same time post BDL (week 2, 4, 6) but not the same age.

### Biochemical measurements

Blood sampling was performed at week 2, 4, and 6, sublingually under isoflurane anaesthesia. Plasma samples were analysed using Reflotron® System (F. Hoffmann-La roche Ltd.) for total bilirubin, and blood ammonium meter (PocketChemTM BA PA-4140) for blood ammonium as markers of biliary obstruction and liver function (p15 BDL animals). In the p21 study (Rackayova et al. 2020) ammonium was measured in plasma using Integra® 400 Plus, therefore the ammonium values are always presented relative to week 2 (week X/ week 2) to minimise the variation when using different measurement devices and so that the two groups p15 vs. p21 (two measurement methods) could be compared. It is well known that ammonium measurements are challenging and sometimes variable in CLD, as such recent guidelines have been published to help improving these measurements (Mallet et al. 2022).

### ^1^H MRS

In vivo brain ^1^H MRS scans were performed at week 2, 4, and 6. During the MR experiments, animals were kept under 1.5-2% isoflurane anaesthesia with respiration rate maintained at 60–70 breaths/min and body temperature at 37.5–38.5 °C.

Measurements were conducted on a horizontal actively shielded 9.4 Tesla system (Magnex Scientific, Oxford, UK) interfaced to a Varian Direct Drive console (Palo Alto, CA, USA) using a home-built quadrature surface coil as a transceiver (17 mm diameter for each loop). The volume of interest (VOI = 2 × 2.8 × 2 mm^3^) for the ^1^H MRS scans was placed in dorsal hippocampus localized on axial and sagittal anatomical T_2_ weighted images (multislice turbo-spin-echo sequence, with repetition time/effective echo time (TR/TE_eff_) = 4000/52 ms, echo train length = 8, field of view = 23 × 23 mm^2^, slice thickness = 1 mm, 2 averages, 256 × 256 image matrix). Hippocampus, as a part of limbic system, was chosen for ^1^H MRS measurements due to known problems with learning and memory in patients with type C HE (Bahceci et al. 2005; Nardelli et al. 2017). The static magnetic field homogeneity was adjusted using first and second order shims by fast, automatic shimming technique by mapping along projections (FAST(EST)MAP) (Gruetter 1993; Gruetter and Tkác 2000), reaching water resonance linewidth 9–10 Hz in the VOI. ^1^H localized spectra were acquired with the ultra-short-echo time spin echo, full intensity acquired localized (SPECIAL) spectroscopy sequence (TE = 2.8 ms, TR = 4 s, 160 averages) (Mlynárik et al. 2006) as previously published (Braissant et al. 2019; Rackayova et al. 2020; Rackayová et al. 2020). Outer volume suppression (OVS) was used to improve signal localization and was interleaved with water signal suppression consisting of RF pulses with variable power and optimized relaxation delays (VAPOR) (Tkáč et al. 1999).

Spectra were fitted and metabolite concentrations were calculated by LCModel (Provencher 2001) and expressed in mmol/kg_ww_ using the unsuppressed water signal from the same VOI as an internal reference. According to literature, brain water content decreases until p28 and stays around 80% afterwards (De Souza and Dobbing 1971; Tkác et al. 2003). Rats in this study were scanned first time 2 weeks after BDL surgery (at p35), therefore brain water was assumed to be 80% during the whole study. The Cramer-Rao lower bounds (CRLB) were used as a reliability measure for the metabolite concentration estimate. Only metabolites with CRLB lower than 30% were considered for further analysis. The LCModel basis-set for spectral fitting contained a spectrum of macromolecules acquired in vivo (Cudalbu et al. 2012; Simicic et al. 2021) and individual metabolites measured in vitro. The ultra-short echo-time MRS allowed the detection of the following 17 metabolites, all included in basis-set: alanine (Ala), ascorbate (Asc), aspartate (Asp), glycerophosphocholine (GPC), phosphocholine (PCho), creatine (Cr), phosphocreatine (PCr), γ-aminobutyric acid (GABA), glutamine (Gln), glutamate (Glu), glutathione (GSH), inositol (Ins), lactate (Lac), N-acetylaspartate (NAA), N-acetylaspartylglutamate (NAAG), phosphoethanolamine (PE) and taurine (Tau). In addition, glucose (Glc), β-hydroxybutyrate (bHB) and scyllo-inositol (Scyllo) signals were included in the basis-set to increase the precision of quantification but their concentrations were not reliably estimated and thus not presented. PCho and GPC were expressed only as tCho (PCho + GPC) due to better accuracy in the estimation of their concentration as a sum. Brain metabolites are expressed in absolute values (mmol/kg_ww_) and in % difference between BDL rats and shams at each time-point in order to account for ongoing development.

### Behavioural tests

Behavioural tests were performed in the animal facility in a silent room at weeks 4 and 6 before MRS scans and blood sampling, always at the same moment of the day. Rats were gently handled 3 times per week by the same person that performed the behavioural tests for habituation. Before the tests, each cage of rats was covered with a tissue and placed in the behavioural room in a separated corner with no visual access to the behavioural tests. The tests started a few minutes after the transfer. Even though the ^1^H MRS experiments were performed in hippocampus as a part of limbic system, due to known problems with learning and memory in HE patients, locomotor activity was assessed in the open field (OF) test to test the presence of fine motor deficits characteristics of type C HE. It involved placing the rat in an open circular arena (100 cm diameter, 32 cm high) (Tzanoulinou et al. 2014). For analysis, the floor was divided into three virtual concentric parts, with a centre zone in the middle of the arena (20 cm diameter), an intermediate zone (60 cm diameter), and an exterior zone comprising the remaining area along the sidewalls. At the start of the test, the animals were placed in the intermediate zone facing the sidewall, and their behaviour was monitored for 10 min using a video camera mounted on the ceiling above the centre of the arena. Different parameters were evaluated with the video tracking system (Noldus Ethovision software 11.5): frequency of crosses, percent time spent, latency to enter (s) in each zone and distance moved (cm) and velocity for the full OF test (cm/s). These parameters were calculated for the entire 10 min of the OF test.

### Statistical methods

All results are presented as mean ± standard deviation (SD, except for behavioural tests presented as mean ± standard error of mean (SEM)). One-way analysis of variance (ANOVA) (Prism 5.03, Graphpad, La Jolla CA USA) with the Bonferroni’s multi-comparisons post-test were used to assess significance (* p < 0.05; ** p < 0.01; *** p < 0.001; **** p < 0.0001) in the measured parameters. If only two sets of data were compared, Student’s t test was used. Pearson correlation analysis was performed on all longitudinally acquired data to test for correlations between the measured parameters.

## Results

### CLD induced by BDL in p15 rats

**Fig. 1:**
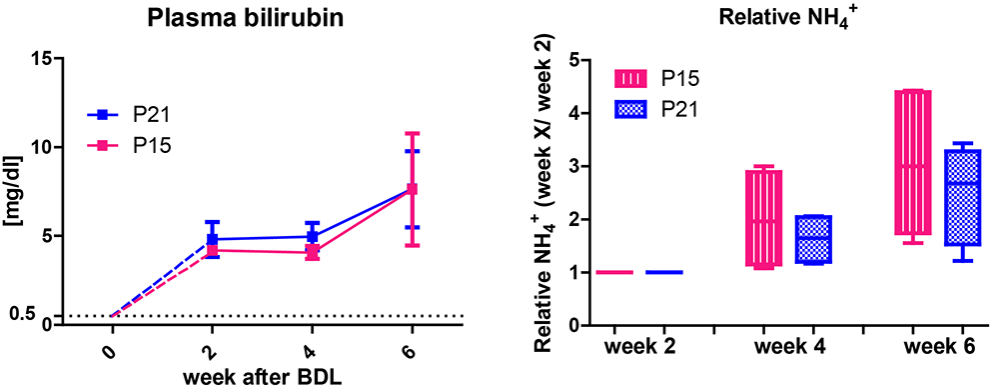
Evolution of bilirubin and ammonium. (Left) Evolution of plasma bilirubin in BDL p15 (in pink) and p21 (in blue) rats during the progression of the disease. Values are expressed in mg/dl. There was no significant difference between groups. (Right) Relative increase in NH_4_^+^ always calculated as week X/ week 2. The data is presented as relative values because the NH_4_^+^ was measured with two different approaches. In case of p21 rats from plasma, Integra ® 400 Plus 16, while for p15 rats it was measured directly from blood using blood ammonium meter (PocketChemTM BA PA-4140) as it was previously mentioned. Note that the relative NH_4_^+^ value does not show the elevation of its absolute concentration usually observed at week 2.

**Plasma bilirubin**, as a marker of liver disease, increased to 4.2 ± 0.3 mg/dl already 2 weeks after BDL surgery and reached 7.6 ± 3.1 mg/dl at week 6. There was no significant difference in plasma bilirubin between BDL p15 and p21 (Fig. 1).

**Relative NH**_**4**_^**+**^ (relative to week 2) in BDL p15 and p21 rats at week 4 and 6 after BDL surgery is shown in Fig. 1 (right). These results show a similar trend of NH_4_^+^ increase between p15 and p21 rats. An approximate ~ 2-fold average increase at week 4 and ~ 3-fold increase at week 6 was observed for p15, while the increase was slightly lower for p21.

### Brain metabolic alterations assessed by in vivo longitudinal ^1^H MRS

**Fig. 2:**
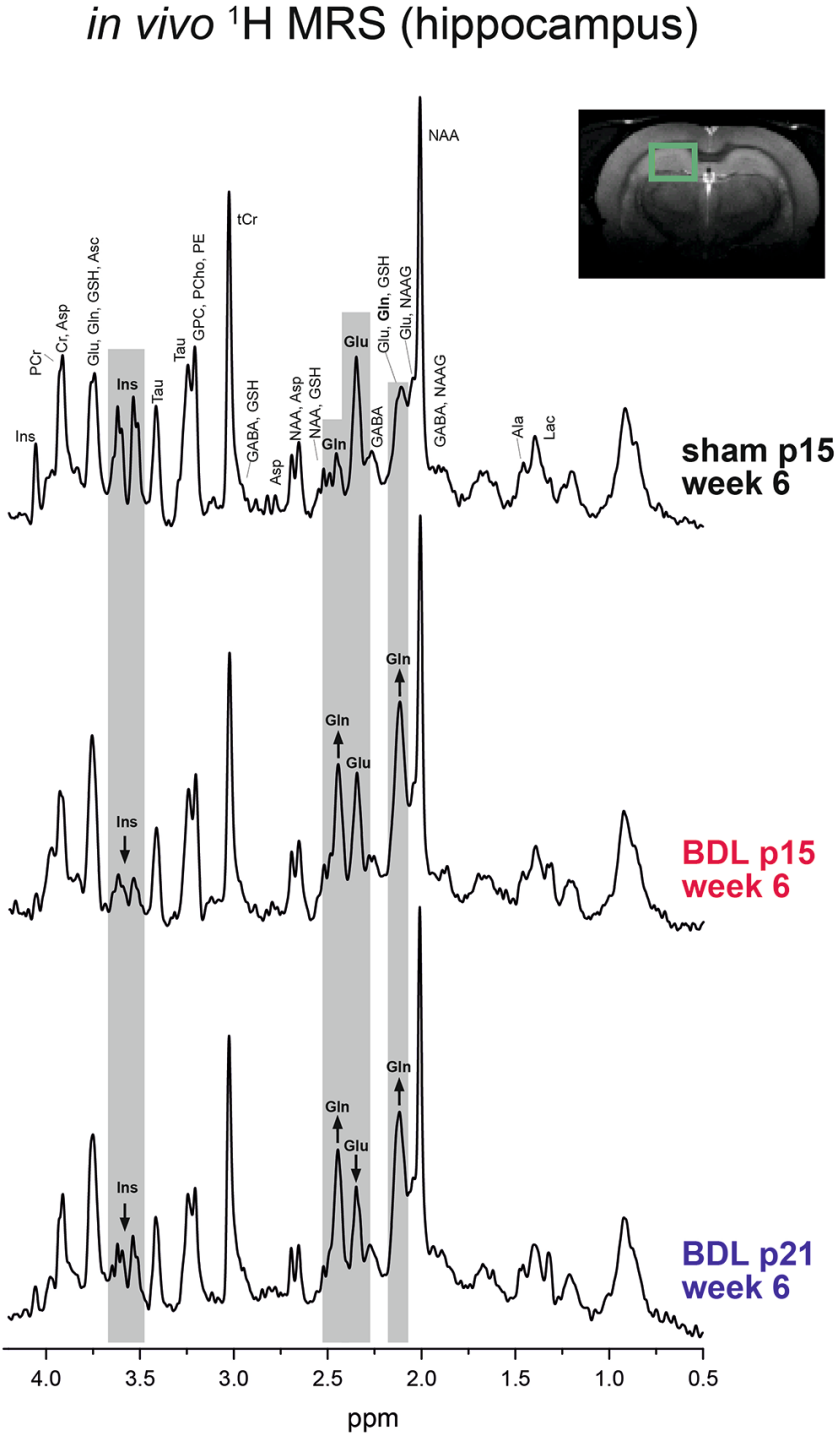
Representative ^1^H MRS brain spectra measured in hippocampus of a sham animal, p15 BDL rat and p21 BDL rat at 6 weeks after sham or BDL surgery. The higher Gln and lower Ins in both p15 and p21 BDL rats compared to the sham animal is visible in the spectra. The p21 BDL rat displayed a lower Glu signal than the p15 BDL rat which is also visible in the spectra.

Figure 2 illustrates the quality of obtained spectra in BDL and sham animals, and some of the most dominant changes (e.g. Gln increase and Ins decrease in both p15 and p21 BDL rats, Glu decrease visible only in p21 BDL rats).

#### Gln and other main organic osmolytes

**Fig. 3:**
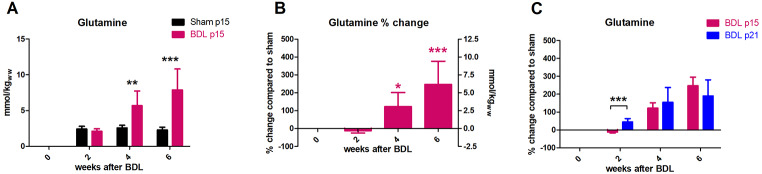
Evolution of brain glutamine. (A) Absolute concentration in mmol/kgww in p15 BDL rats (pink) and sham (black). (B) Percental change in p15 BDL compared to sham rats at corresponding age. (C) Comparison between percental change in p15 BDL (pink) and in p21 BDL rats (blue) compared to their sham at corresponding age. *(pink) is compared to change at week 2.

Two weeks after BDL surgery, p15 BDL rats did not show any increase in **Gln**, in contrast to p21 BDL rats that had significantly higher Gln at week 2 after BDL surgery. Gln increase, for p15 BDL rats, was significant at week 4 (122 ± 79%, p < 0.01) and further increased at week 6 after BDL (247 ± 129%, p < 0.001) (Fig. 3A,B). The difference between p15 and p21 BDL rats in Gln increase at week 2 was significant (p < 0.001). However, at week 4 and 6 there was no statistical difference in the Gln increase between p15 and p21 BDL rats, even though p15 rats showed a slightly higher Gln increase in % change at week 6 (247 ± 129% for p15 compared to 190 ± 90% for p21, Fig. 3C).

**Fig. 4:**
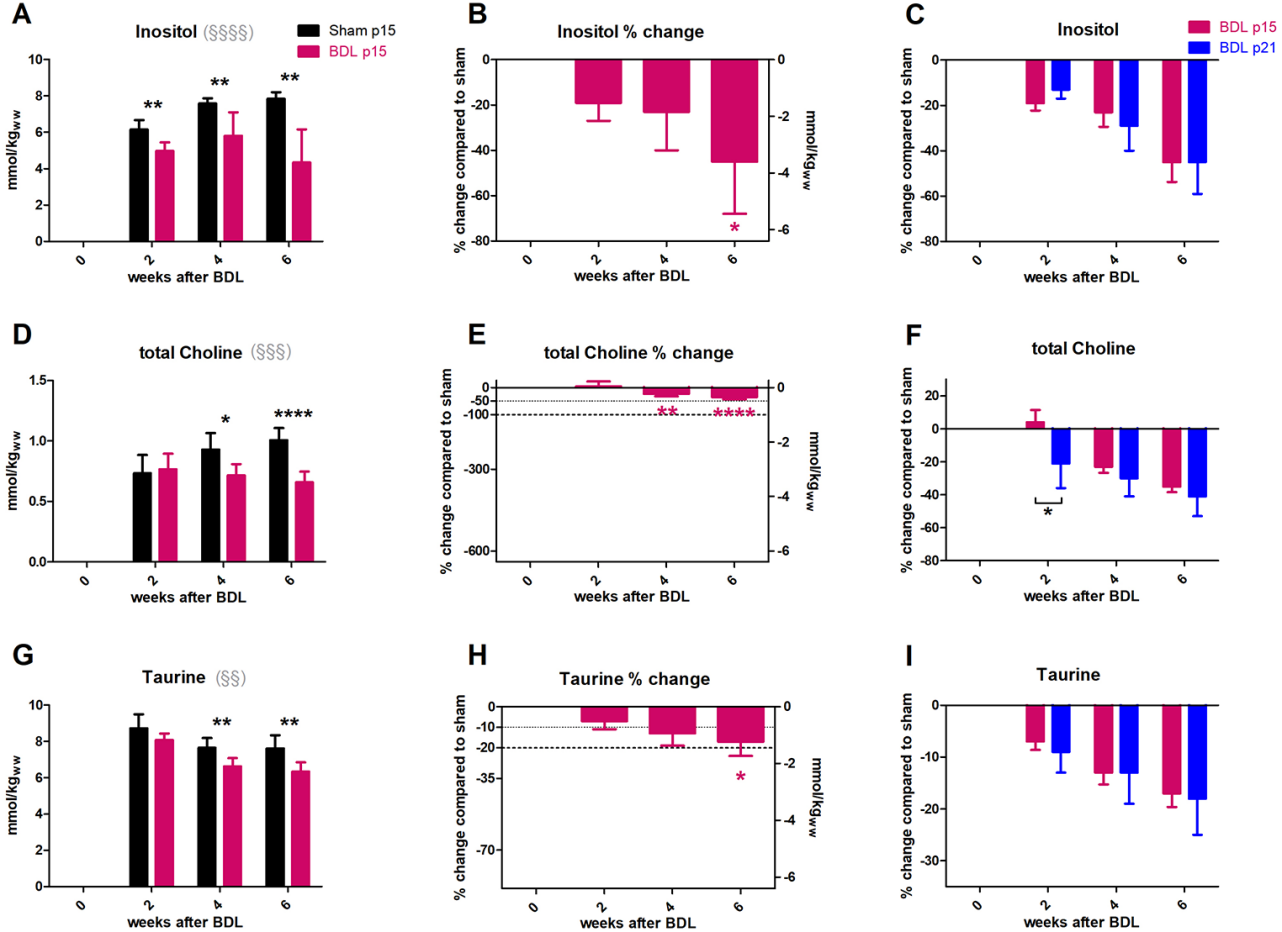
Evolution of brain organic osmolytes. (A, D, G) Absolute concentration in mmol/kgww in p15 BDL (pink) and sham (black). (B,E,H) Percental change in p15 BDL compared to sham at corresponding age. (C,F,I) Comparison between percental change in p15 BDL (pink) and in p21 BDL (blue) compared to their sham at corresponding age. *(pink) is compared to change at week 2; § indicates significant change in sham animals due to ongoing brain development in agreement with (Račkayová et al. 2021). Note: the scale of right y-axis in the middle column in mmol/kg_ww_ is set at the same range for all metabolites. This is for better visual comparison of their contribution to the osmoregulation.

Figure 4 displays changes in the metabolites considered as main brain organic osmolytes. **Ins** decreased in p15 BDL rats significantly at week 2 after BDL surgery (-19 ± 8%, p < 0.01) and reached a decrease of -45 ± 23% (p < 0.01) at 6 weeks after BDL (Fig. 4A,B). There was no difference between Ins decrease in p15 and p21 BDL rats at any time-point (Fig. 4C).

**tCho** showed a significant decrease 4 weeks after BDL (-23 ± 10%, p < 0.05) decreasing further to -35 ± 9% (p < 0.001) at week 6 (Fig. 4D,E). The response of tCho in p15 and p21 BDL was significantly different at week 2 while there was no difference in tCho decrease at the other time-points (Fig. 3F). **Tau** decreased significantly 4 weeks after BDL (-13 ± 6%, p < 0.01), reaching − 17 ± 7% (p < 0.01) at week 6 (Fig. 4G,H). There was no difference in Tau decrease between p15 and p21 BDL rats (Fig. 4I).

**Fig. 5:**
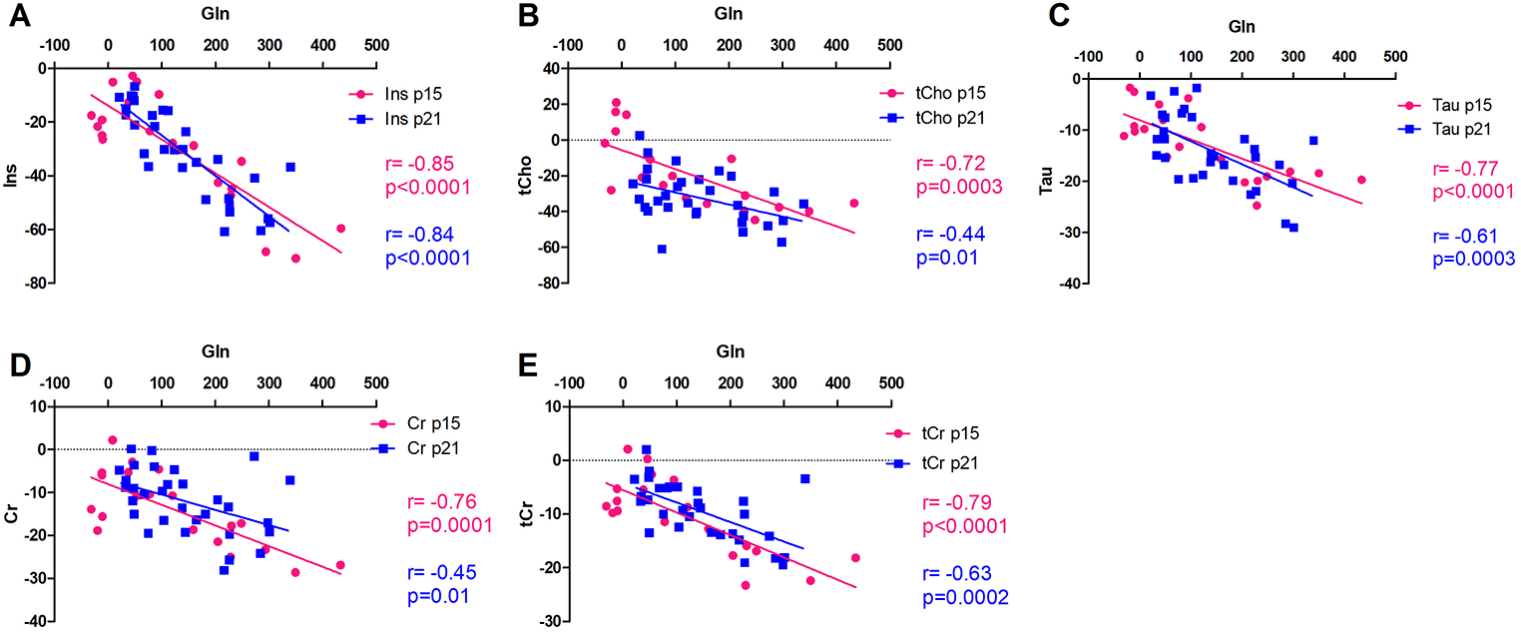
Correlations between brain glutamine and the other brain osmolytes. Correlation between change in Gln and change in other osmolytes (Ins, tCho, Tau, Cr, tCr). P15 BDL are presented in pink and p21 BDL in blue.

P15 BDL rats showed strong correlations between the increase in Gln and the decrease in all osmolytes (Ins, tCho, Tau, Cr, tCr) (Fig. 5). This relation between Gln and osmolytes was similar to that in p21 for Ins, Tau and tCr but p15 BDL displayed a steeper slope for tCho, despite the fact that in p15 BDL rats Gln increased significantly 4 weeks after BDL surgery compared to a significant increase at week 2 in p21 BDL rats (Rackayova et al. 2020).

#### Energy metabolites and metabolic stress

**Fig. 6:**
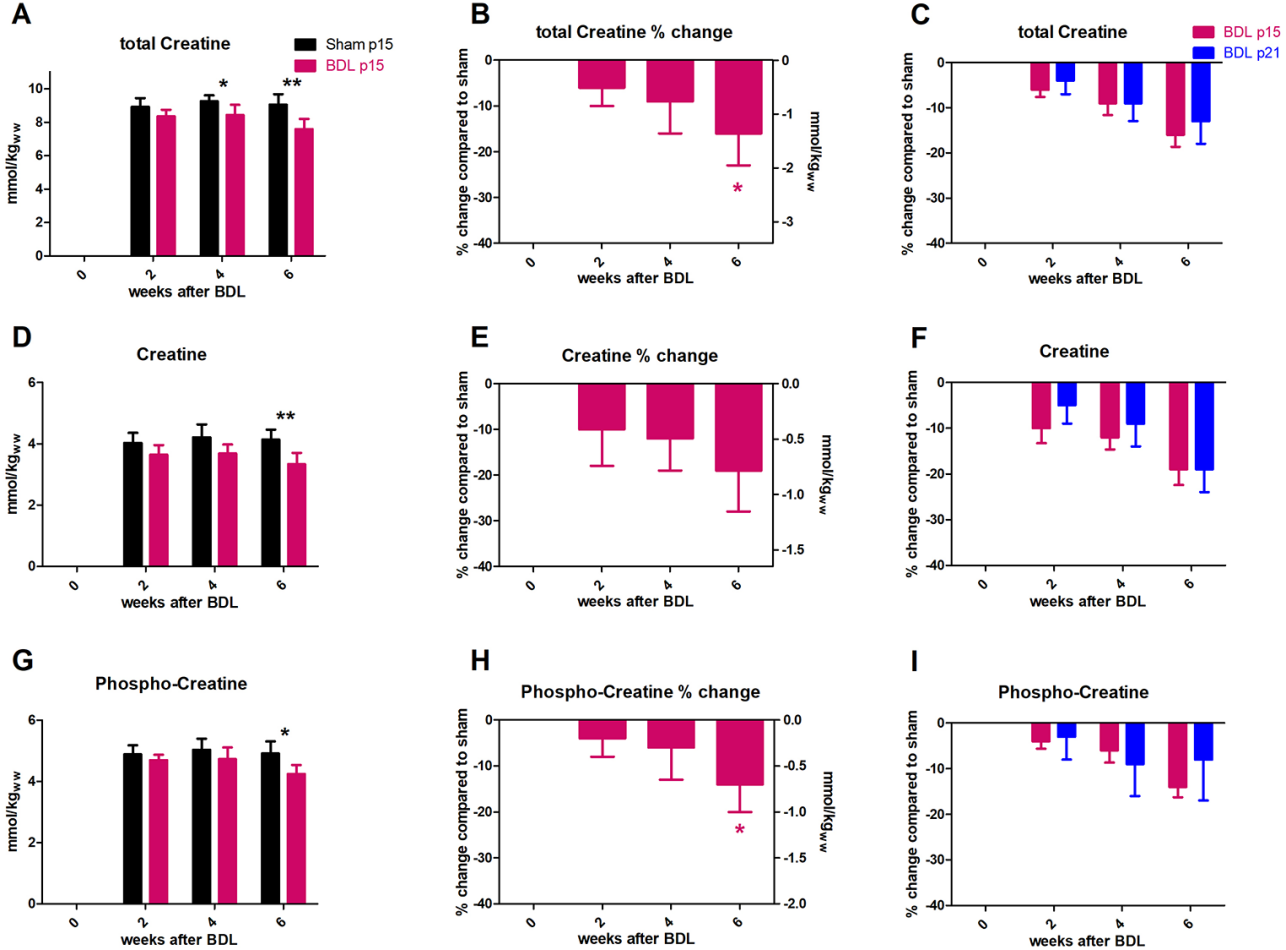
Evolution of brain creatines. (A, D, G) Absolute concentration in mmol/kg_ww_ in p15 BDL (pink) and sham (black). (B,E,H) Percental change in p15 BDL compared to sham at corresponding age. (C,F,I) Comparison between percental change in p15 BDL (pink) and in p21 BDL (blue) compared to their sham at corresponding age. *(pink) is compared to change at week 2.

**Cr** and **PCr**, which are considered both as being involved in energy metabolism and Cr also in osmoregulation (Heilig et al. 1989; Bothwell et al. 2002; Hanna-El-Daher and Braissant 2016), decreased significantly at week 6 after BDL surgery. Cr decreased by -19 ± 9% (p < 0.01) and PCr by -14 ± 6% (p < 0.05) (Fig. 6D,E and G,H).There was no significant difference in Cr or PCr decrease between p15 and p21 BDL rats throughout the study (Fig. 6F,I). The sum, **tCr** (Cr + PCr) decreased significantly in p15 BDL rats week 4 (-9 ± 7%, p < 0.05) and further to -16 ± 7% (p < 0.01) at week 6 (Fig. 6A,B) with no significant difference in its decrease between p15 and p21 BDL rats (Fig. 6C).

**Fig. 7:**
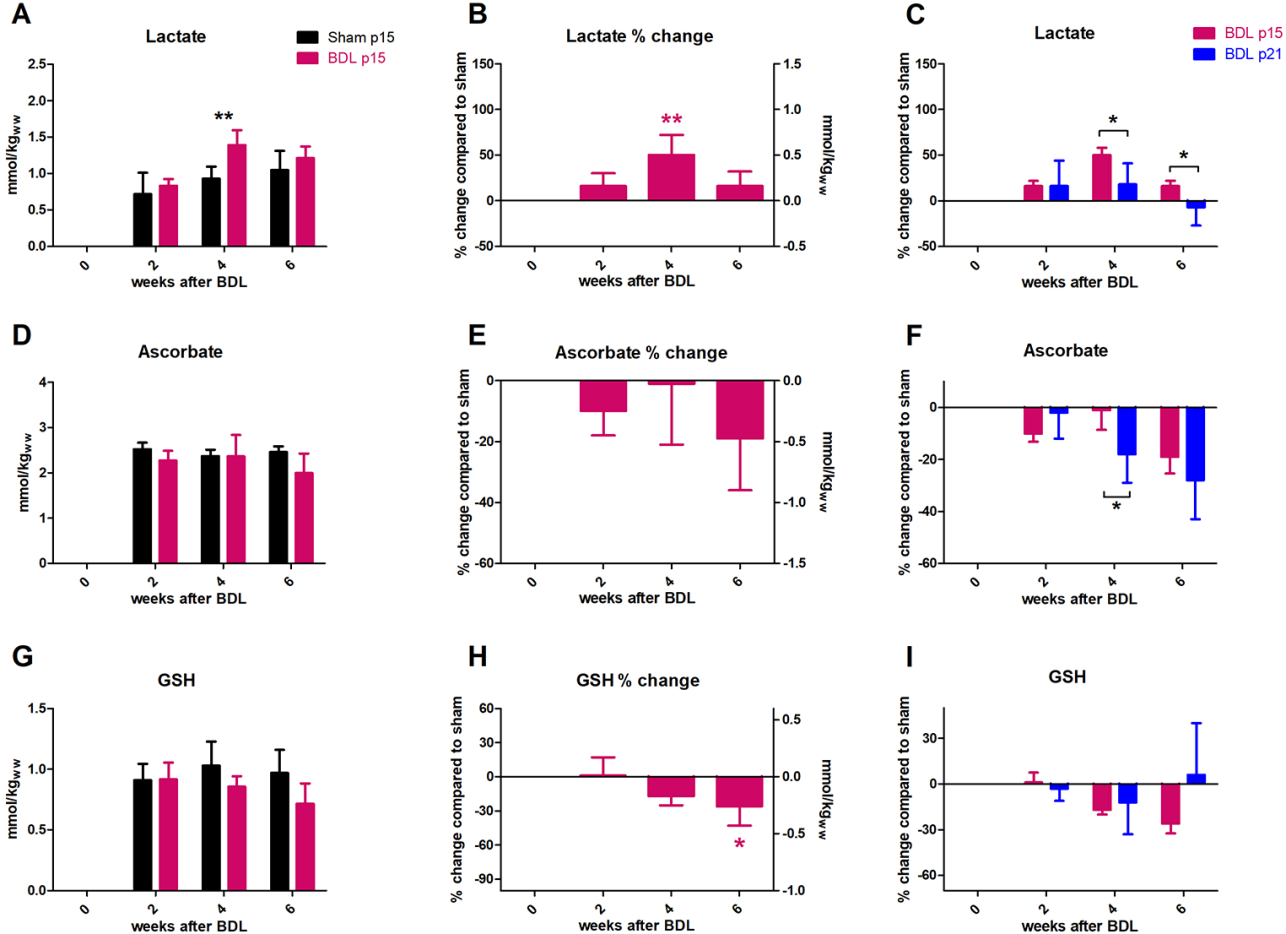
Evolution of brain lactate and antioxidants. (A, D, G) Absolute concentration in mmol/kg_ww_ in p15 BDL (pink) and sham (black). (B, E, H) Percental change in p15 BDL compared to sham at corresponding age. (C, F, I) Comparison between percental change in p15 BDL (pink) and in p21 BDL (blue) compared to their sham at corresponding age. *(pink) is compared to change at week 2.

**Lac** levels were significantly increased in p15 BDL brain at week 4 after BDL surgery (50 ± 22%, p < 0.01) and remained high at week 6 (16 ± 16%) without reaching significance (Fig. 7A,B). Lac increase was significantly higher than in p21 BDL rats at weeks 4 and 6 (Fig. 7C).

#### Antioxidants

**Asc** stayed stable until week 4 and showed a decrease at week 6 (-19 ± 17%) without reaching significance (Fig. 7D,E). p21 BDL showed a significant decrease already from week 4 and the difference between Asc decrease in p21 and p15 was significant at week 4. There was no significant difference in Asc decrease between p15 and p21 BDL rats at week 6 after BDL surgery.

**GSH** showed a decrease of -26 ± 17% at 6 weeks after BDL (Fig. 7G,H), significant compared to its change at week 2 after BDL surgery but insignificant when compared to shams. GSH decrease in p15 BDL rats, when compared to p21 BDL, was more pronounced and became significantly different at week 6 (Fig. 7I).

#### Neurotransmitters

**Fig. 8:**
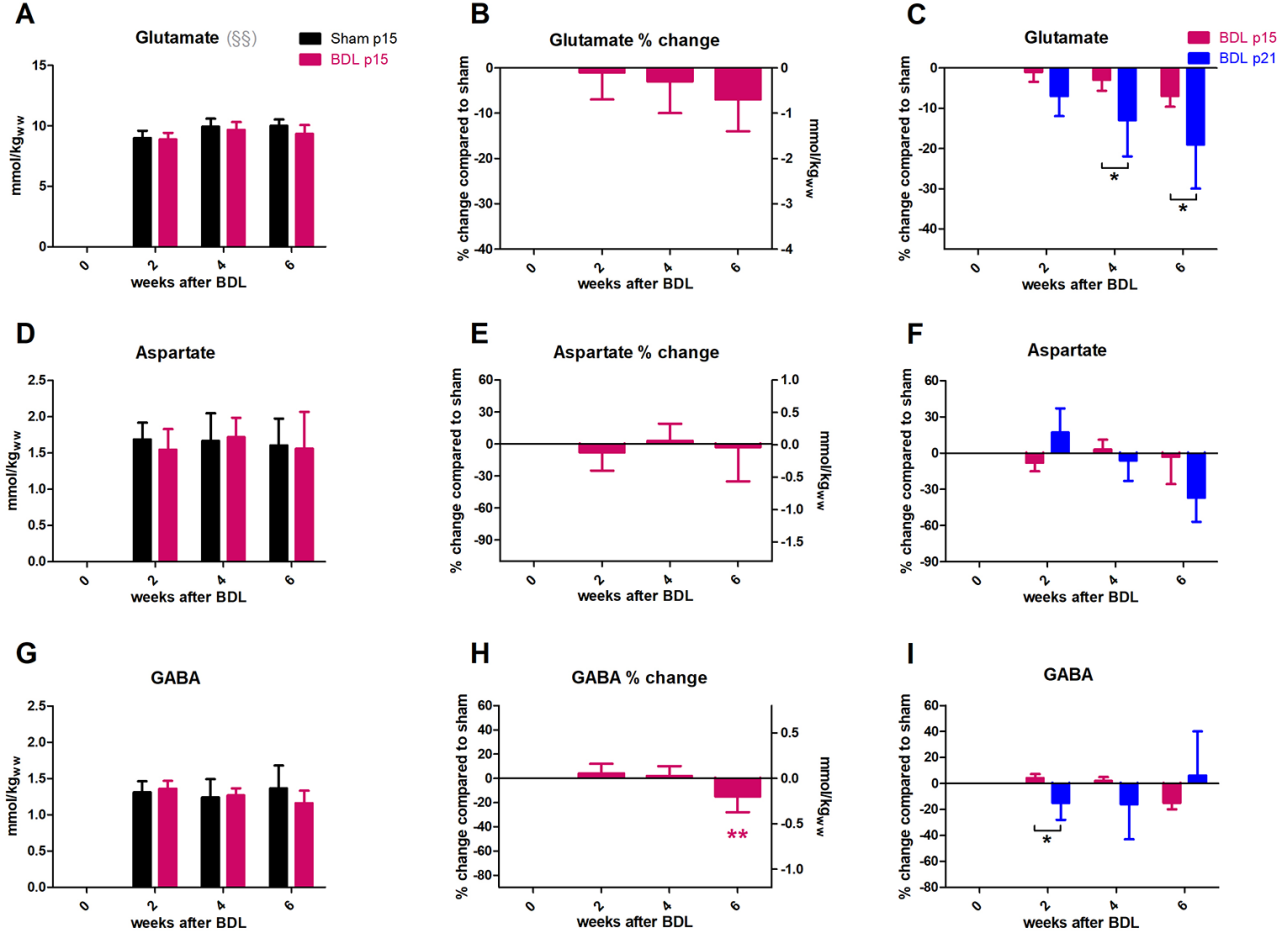
Evolution of neurotransmitters. (A, D, G) Absolute concentration in mmol/kg_ww_ in p15 BDL (pink) and sham (black). (B, E, H) Percental change in p15 BDL compared to sham at corresponding age. (C, F, I) Comparison between percental change in p15 BDL (pink) and in p21 BDL (blue) compared to their sham at corresponding age *(pink) is compared to change at week 2; § indicates significant change in sham animals due to ongoing brain development in agreement with (Račkayová et al. 2020).

None of the neurotransmitters (Glu, Asp, GABA) showed any significant decrease throughout the study in p15 BDL rats. **Glu** showed an insignificant decrease of -7 ± 7% at 6 weeks after BDL (Fig. 8A,B) in contrast to p21 BDL rats with significantly more important decrease in Glu at weeks 4 and 6 after BDL (Fig. 8C). **Asp** did not show any change throughout the study. **GABA** reached − 15 ± 13% at week 6 (Fig. 8G,H), insignificant compared to sham but significant compared to its change at week 2. The difference in GABA decrease between p21 and p15 BDL rats was significant at 2 weeks after BDL (Fig. 8I).

**Fig. 9:**
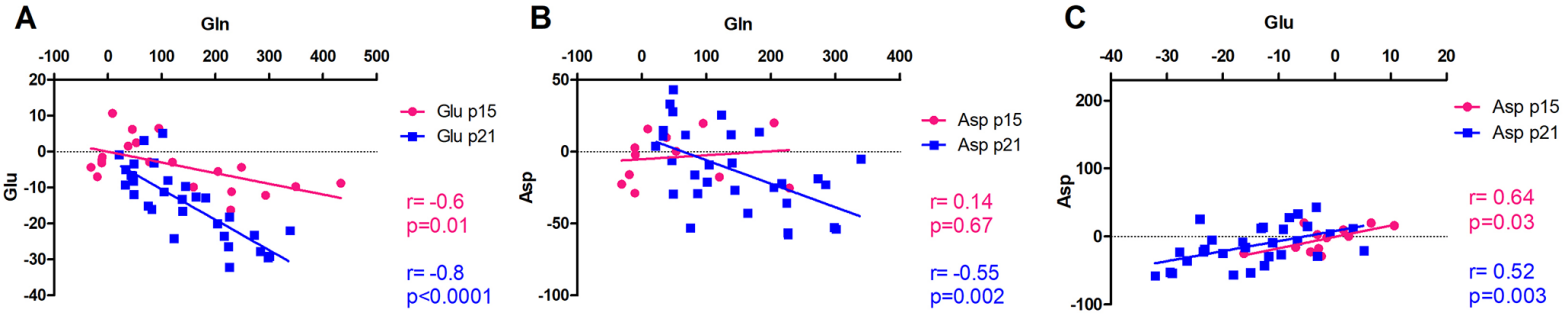
Correlations between metabolites involved in neurotransmission. (A,B) Correlation between change in Gln and change in neurotransmitters (Glu, Asp). (C) Correlation between change in Glu and change in Asp. P15 BDL are presented in pink and p21 BDL in blue.

The correlations between Gln increase and decrease in neurotransmitters were much weaker in p15 BDL rats compared to p21 BDL rats. The correlation between Gln and Glu was present in p15 BDL rats but the slope was not as steep as in p21 BDL rats (Fig. 9A). There was no correlation between Gln and Asp in p15 BDL rats, in contrast to p21 BDL (Fig. 9B). Moreover, there was no correlation between Gln and GABA neither in p21 nor p15 BDL rats (data not shown). However, the change in Glu and Asp correlated well in both p15 and p21 BDL rats (Fig. 9C).

#### Stable metabolites during CLD

Ala, NAA, NAAG and PE did not show any significant difference between p15 BDL rats and their shams throughout the study.

### Behavioural tests

**Fig. 10:**
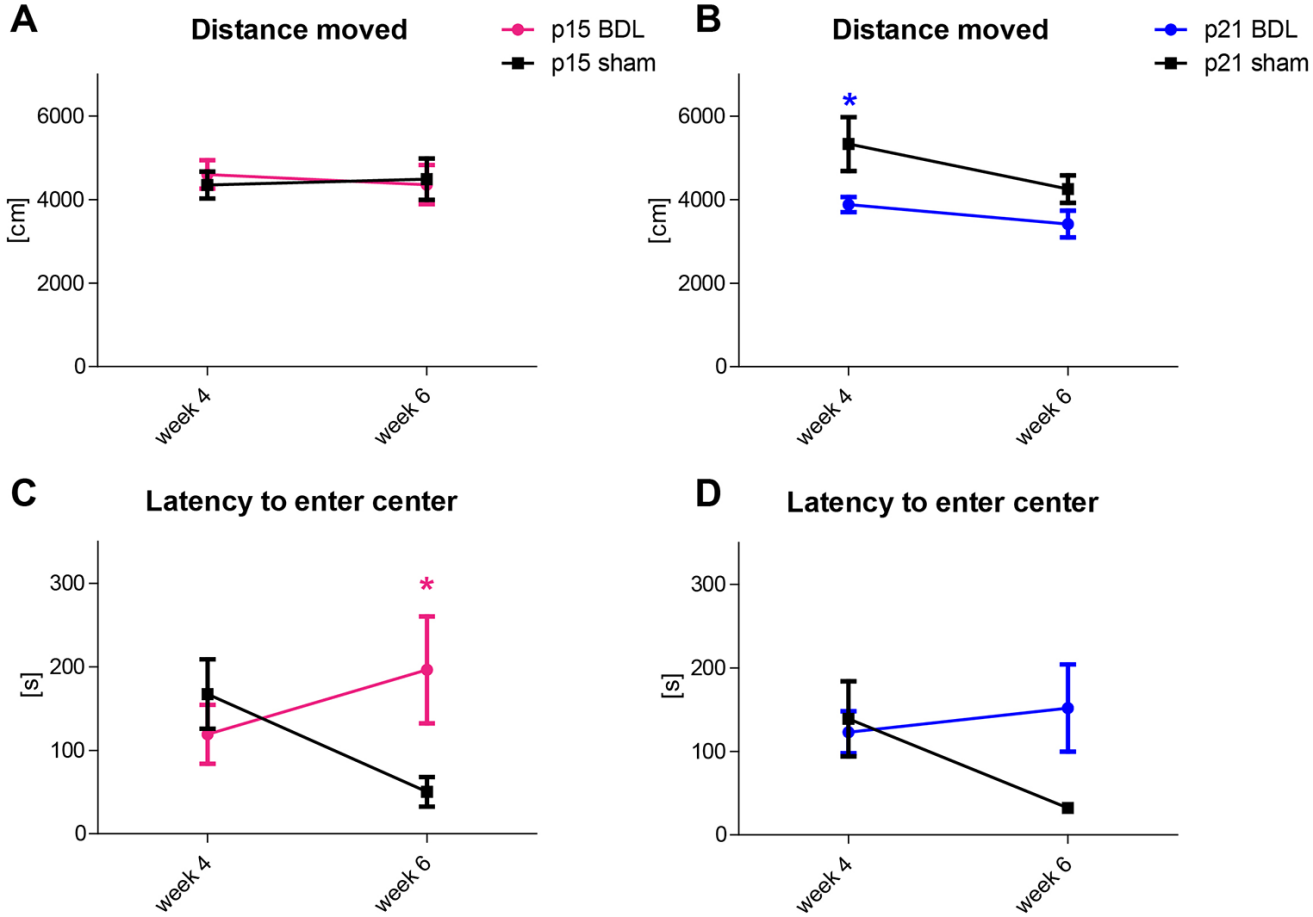
Performance in behavioural tests. (A, B) Distance moved during 10 min Open Field test. (C, D) Latency to enter the center of arena for the first time during the Open Field test. P15 BDL (n = 7) are presented in pink and p21 (n = 9) BDL in blue.

In the Open Field test, there was no difference in the distance moved between p15 BDL and their shams (Fig. 10A), in contrast to p21 BDL rats (Fig. 10B). But p15 BDL rats showed a significant delay in first enter to the centre of arena at 6 weeks after BDL (Fig. 10C). P21 BDL rats showed only a trend of increased delay to enter the centre of arena (Fig. 10D).

## Discussion

The present work showed for the first time that when CLD was acquired in rats at p15, the rats presented the typical signs of CLD, i.e. rise in plasma bilirubin and ammonium, and developed the characteristic brain metabolic changes associated with type C HE namely an increase in brain Gln which correlated with the decrease in the other main osmolytes (Ins, Tau, tCho, Cr). When compared to the group of rats that acquired CLD at p21, p15 rats did not show any significant difference in plasma biochemistry (bilirubin and ammonium) but did display a delayed increase in brain Gln and decrease in tCho. In addition, the changes in neurotransmitters were milder than in p21 rats. On the other hand, p15 rats showed an earlier increase in brain Lac and a different antioxidant response than p21 rats, and a decrease in exploratory behaviour. The cellular and enzymatic underpinnings remain unknown, yet these differences suggest that the brain of younger pups does respond differently to the metabolic insults of CLD. Whether this is due to maturing enzymes and metabolic pathways remains to be elucidated. Nonetheless, these novel findings raise the question of whether similar changes might exist in humans but are missed owing to ^1^H MRS methodological limitations in field strength in clinical magnets.

P15 BDL rats started to develop CLD during earlier phases of brain development than p21 BDL rats. An increase of NAA has been observed by ^1^H MRS in humans during infancy and early childhood and connected with the increase in the number of neurons, formation of dendritic arborizations and synaptic connections (Pouwels et al. 1999). In the rat brain NAA has been shown to increase in hippocampus between p15 and p21 and to stabilize thereafter (Tkác et al. 2003), in agreement with our shams. Even though the hippocampus grows in mass through p70, the non-neuronal to neuronal cell ratio increases only until p20 (Bandeira et al. 2009). Although neurogenesis occurs mainly during the embryonal development in rats (Wiggins 1986), the hippocampal dentate gyrus undergoes quite late neurogenesis and dentate granule cells are formed until p19 (Bayer et al. 1993). This period of time coincides with the first week after BDL in p15 rats. Similarly, rat brain synapses seem to mature until p21 (Jacobson 1991). Also, an important part of gliogenesis (especially astrocytes and oligodendrocytes) takes place after birth and continues until the weaning period (Rice and Barone Jr 2000). Therefore, there are distinctive differences in the neurodevelopmental processes in p15 and p21 rats. Thus, it is interesting to consider that neurocognitive outcomes are in part influenced by time of CLD onset and cumulative exposure to liver derived metabolites and other compounds. Not only does time of CLD onset have the potential to influence neurodevelopmental processes, but the earlier the onset, the longer the exposure and higher the risk of influencing multiple, sequential neurodevelopmental events.

### Ammonium and brain glutamine

Differential Gln increase between p15 and p21 rats might be explained by the maturation of glutamine synthetase (GS) – the enzyme that allows astrocytes to detoxify ammonium by condensing Glu and ammonium. GS seems not to be fully active until p26 (Bayer and McMurray 1967), possibly explaining why in rats that underwent BDL surgery at p15 Gln did not increase at week 2 (corresponding to p29). Therefore, at this stage, the effect of ammonium might be a direct toxic effect rather than through Gln and osmotic stress. Finally, a role of maturation of the blood-brain barrier cannot be excluded (Hirase et al. 1997; Cagnon and Braissant 2007).

### Osmotic response

P15 and p21 BDL rats showed a very similar decrease in main organic brain osmolytes, except for tCho. At 2 weeks after BDL surgery, Ins decreased significantly and Tau, Cr showed a trend of decrease even though Gln still did not rise. Whether the decrease in these metabolites was driven by direct osmotic effects of ammonium, or other mechanisms were involved needs further investigation.

#### Inositol

Similar to Tau, Ins showed a decrease in p15 and p21 BDL rats during the progression of the liver disease. This strong decrease in Ins starting from week 2, probably as osmotic compensation, can have an important impact on the brain development. Myo-inositol is a main source of inositol for phosphatidyl-inositol and phosphoinositides, playing an important role in membrane metabolism and signalling as well in the formation of myelin. Ins has a very high metabolic turnover in living systems which can be altered in pathologies where Ins pool is reduced (Greene et al. 1975; Zhu and Eichberg 1990). In addition, inositol trisphosphate (IP_3_) – Ca^2+^ signalling pathways in the CNS are contributing to the synaptic plasticity and thus learning and memory (Berridge 1998). IP_3_ receptors are also located in the dendritic spines of Purkinje cells in the cerebellum and are responsible for motor learning (Sharp et al. 1993). Therefore, type C HE symptoms such as memory problems and motor deficits could be potentially linked with Ins decrease, especially if the Ins decrease occurs early in life at a time of intense synaptic development. Moreover, in physiological conditions, Ins should increase in the developing brain, as has been previously shown by us and others (Tkác et al. 2003; Račkayová et al. 2020) and also observed for the sham animals in the present study. Therefore, it can be suggested that a depletion of Ins might be deleterious for membrane formation, another way in which CLD impacts the developing brain.

#### Total choline

tCho showed a later decrease in the p15 rats than in p21 BDL rats (at week 2). Whether tCho decreased as a part of an osmoregulatory process or as a result of impaired phospholipid metabolism is not clear. However, it could be argued that its decreased concentration in the brain during development could significantly impact membrane metabolism. PCho and GPC are the main sources of choline for phosphatidyl-choline (Amenta et al. 2001) and phosphatidyl-choline together with phosphatidyl-ethanolamine account for 90% of membrane phospholipids (Vance 2008). In addition, the decrease in tCho but no change in PE in the brain of BDL rats will change the choline-ethanolamine ratio. This might in turn influence membrane properties as phosphatidyl-choline is located predominantly on the extracellular layer of neural plasma membrane and phosphatidyl-ethanolamine on the intracellular layer (Ikeda et al. 2006; Deleke 2007; Harper et al. 2014). It is well known that tCho decreases in the healthy developing rat brain until p28 (Tkác et al. 2003) and that it increases after p29 as shown in the present study and in agreement with previously published work (Račkayová et al. 2021). This might explain the different behaviour of tCho in p15 and p21 BDL rats.

Importantly, it has been shown that the consequences of a decrease in brain phospholipids caused by malnutrition in young rats could not be recovered by rehabilitation (Reddy and Sastry 1978). Therefore similarly, the decrease of tCho in BDL rats, as a metabolite involved in phospholipid metabolism, could potentially have long-term effects such as contributing to the residual neurocognitive deficits in children even after liver transplantation (Stewart et al. 1991; Caudle et al. 2010, 2012; Sorensen et al. 2014).

#### Taurine

As mentioned, Tau showed a tendency to decrease at week 2 that became significant 4 weeks after BDL in p15 BDL rats compared to their shams, and this mimicked the observations in p21 BDL rats. The concentration of Tau physiologically decreases during brain development (Huxtable 1992), something which we observed in the shams and in our previous study (Račkayová et al. 2021). It has been hypothesized that this decrease may reflect the loss of maternal Tau (Agrawal et al. 1971). The decrease of Tau in BDL rats was more drastic than the one physiologically occurring in shams. Tau has an important osmoregulatory function therefore it is highly possible that its decrease is primarily linked with osmoregulation. However, Tau in the infant brain is considered cytoprotective possibly assuming an antioxidant role. Therefore, a supraphysiological decrease in Tau may deprive the developing brain from this protection at a time when it would crucially need it (Pasantes-Morales and Hernández-Benítez 2010).

### Energy metabolism

Cr and PCr showed a similar decrease in p15 and p21 rats at all time-points. As p21 rats, p15 rats showed a quick decrease in Cr already 2 weeks after BDL surgery, probably as osmoregulatory process. However, a long-term decrease in Cr and consequently in tCr over 6 weeks after BDL surgery might be due to its impaired synthesis, as ammonium was shown to inhibit the first of the two enzymes responsible for Cr synthesis (arginine:glycine amidinotransferase (AGAT)) in developing brain cell 3D cultures (Braissant et al. 2008). Such a significant decrease in both metabolites can further create an impairment in energy metabolism.

Lac showed a very different pattern in p15 BDL than in p21 BDL rats. In p15 BDL rats, Lac increased significantly at 4 weeks after BDL surgery and stayed significantly higher than in p21 BDL rats also at week 6. Of note, the p21 rats showed an increase of Lac at week 8 post-BDL reaching similar values to p15 pups at 6 weeks post-BDL. This different behaviour can be explained by the fact that p21 rats seemed visually less sick at 6 weeks post-BDL and survived longer.

### Antioxidants

P15 BDL rats showed a different pattern in the antioxidant response to CLD than p21 BDL rats. In p15 BDL rats, Asc decrease was non-significant and smaller than in p21 BDL rats, showing a more important decrease only later in the disease (6 weeks after BDL). In p21 BDL rats, Asc showed a continuous decrease, significantly stronger at week 4 after BDL surgery than for p15. On the other hand, GSH in p15 BDL rats decreased gradually showing a more pronounced change than GSH in the brain of p21 BDL rats. GSH in p21 BDL rats stayed stable until week 6.

As Asc is known to be predominantly in neurons and GSH to be in glial cells (Raps et al. 1989; Makar et al. 1994; Rice and Russo-Menna 1998), different antioxidant response in p15 and p21 BDL brain might be an indication that different cell types are more affected in p15 compared to p21 BDL rats. Taken together, a more pronounced decrease in GSH in p15 BDL rats than p21 with a rising ratio of non-neuronal cells to neuronal cells until p21 (Bandeira et al. 2009), combined with an ongoing gliogenesis (Rice and Barone Jr 2000) and with a phase of strong myelination (Jacobson 1963; Wiggins 1986; Meier et al. 2004), could suggest that the glial cells might be more affected by the disease in p15 BDL rats than in p21. On the other hand, the stronger Asc decrease as well as a more significant decrease in neurotransmitters in p21 BDL rats compared to p15 BDL rats can indicate a stronger effect on neurons. In addition, significantly higher levels of Asc in the brain of p15 BDL than in p21 BDL rats can also have a protective effect on glutamatergic neurotransmission, as Asc has been linked to Glu release and uptake (Rice 2000).

### Neurotransmitters

Neurotransmitters were not strongly affected in p15 BDL rats, especially compared to p21. Glu showed a non-significant trend of decrease, Asp did not change and GABA decreased significantly only at week 6.

We previously suspected that Glu decrease could be considered as a consequence of increased Gln synthesis. Therefore, a smaller Glu decrease in p15 compared to p21 BDL rats might be related to the delayed increase in Gln in p15 BDL rats. However, another reason could be a later maturation of brain enzymatic activities involved in amino acid metabolism. Many enzymes importantly increase their activities between p10-p20, GS even until p26 (Bayer and McMurray 1967; Agrawal et al. 1971), and stabilize only afterwards. This can influence the coupling between Gln increase and decrease in neurotransmitters. Nevertheless, Asp and Glu decrease seemed to be closely related in both p15 and p21 BDL rats demonstrated by their tight correlation in both groups of rats (Fig. 9C). It is unclear whether the differences between the p15 and p21 neurotransmitter profiles are indicative of differences in enzymatic maturation or of protective mechanisms is the younger brain.

Although some of the neurometabolic differences are very subtle between the p15 and p21 BDL rats, p15 BDL rats were visually sicker than p21 BDL rats and performed less well on behavioural tests. While their motor activity was similar when compared to their shams (in contrast to p21 BDL rats), p15 BDL rats showed significantly increased latency to enter the centre of Open Field arena during the test. Indeed, this difference in the decrease of motor activity in p21 BDL rats and decrease in the exploratory behavioural in p15 BDL may simply be related to behavioural development. Exploratory activity dominates over locomotor activity between p20 and p30 and they interchange afterwards (Bâ and Seri 1995). Our behavioural tests are performed at p30, but p15 BDL rats were developing CLD during the window of the development of exploratory behaviour what might have affected this feature in p15 BDL rats more than in p21 BDL rats.

## Limitations

It is important to note that the goal of this study was to analyze the neurometabolic response of the brain to CLD depending on the time/age of the disease onset. Therefore, the animals were always scanned at the same timepoint during disease development (i.e. 2, 4 and 6 weeks after BDL surgery). However, this approach did not allow a direct comparison of the animals at the same age, meaning that p15 BDL operated rats are at p29 (p43, p57) at week 2 (4, 6) after surgery while p21 BDL operated rats are at p35 (p49, p63) at week 2 (4, 6) after surgery.

## Conclusion

In conclusion, we showed that the neurometabolic changes of rats that developed CLD at p15 display many similarities with p21 BDL rats and even adult rats, for example in the osmoregulatory response to CLD. There are however a few notable differences such as a less rapid increase in Gln and decrease in tCho, Asc concentrations; and a more significant and earlier Lac increase and GSH decrease. Importantly, Gln concentrations were slightly higher in p15 rats at the end of the study. Effect of the disease on the concentrations of neurotransmitters was minor in p15 BDL rats. These findings offer tentative pointers as to which neurodevelopmental processes may be impacted. Ultimately it is the fine balance of how osmotic shifts and molecular fluxes impact membrane formation, neuro- and synaptogenesis, as well as the proliferation and differentiation of other cell types which will lead to the behavioral or neurological or neurocognitive phenotype. Here we compared neurometabolic changes between p15 and p21 BDL rats in the hippocampus. Knowing that brain regions are enriched in different molecules to perform their different functions, it seems reasonable to extrapolate that different brain regions will respond differently to the metabolic insults of CLD not just longitudinally but also spatially, something important to explore if we are to progress in our understanding of the vast phenotypic spectrum of type C HE.

## Data Availability

The data is available on the following repository 10.5281/zenodo.7406309.
